# The Effects of Obesity and Mobility Disability in Access to Breast and Cervical Cancer Screening in France: Results from the National Health and Disability Survey

**DOI:** 10.1371/journal.pone.0104901

**Published:** 2014-08-18

**Authors:** Clémence Bussière, Jonathan Sicsic, Nathalie Pelletier-Fleury

**Affiliations:** Cermes3 – Inserm U988, Villejuif, France; State University of Maringá/Universidade Estadual de Maringá, Brazil

## Abstract

**Objectives:**

We aimed to disentangle the effects of obesity and mobility limitation on cervical and breast cancer screening among community dwelling women.

**Methods:**

The data source was the French national *Health and Disability Survey - Household Section*, 2008. The Body Mass Index (BMI) was used to categorize obesity status. We constructed a continuous score of mobility limitations to assess the severity of disability (Cronbach's alpha = 0.84). Logistic regressions were performed to examine the association between obesity, mobility limitations and the use of Pap test (n = 8 133) and the use of mammography (n = 7 561). Adjusted odds ratios were calculated (AOR). Interaction terms between obesity and the disability score were included in models testing for effect modifications.

**Results:**

Compared with non-obese women, the odds of having a Pap test in the past 3 years was 24% lower in obese women (AOR = 0.76; 95% CI: 0.65 to 0.89), the odds of having a mammogram in the past 2 years was 23% lower (AOR = 0.77; 95% CI: 0.66 to 0.91). Each time the disability score was 5 points higher, the odds of having a Pap test decreases by 20% (AOR = 0.96; 95% CI: 0.94 to 0.98), the odds of having a mammogram decreases by 25% (AOR = 0.95; 95% CI: 0.94 to 0.97). There was no significant interaction between obesity and disability score.

**Conclusion:**

Obesity and mobility limitation are independently associated with a lower likelihood of cervical and breast cancer screening. Protective outreach and follow-up are necessary to reduce inequalities and thus to reduce health disparities in these vulnerable and high-risk populations of obese women with disabilities.

## Introduction

Cancer is a leading cause of death worldwide and accounted for 8.2 million deaths in 2012 [1]. Breast and cervical cancers are among the most common cancers in women and are curable if detected early [2]. For this reason, the mammogram and Pap test are widely offered as part of national cancer screening programs in developed countries. Unfortunately, participation in screening is variable [3], [4], even in health systems with adequate resources [5]. Disadvantages related to socio-economic factors and health variables are typically associated with inequalities in access to preventive care [6]–[9].

Emerging evidence links both obesity and disability with a relatively low likelihood of cancer screening [10]–[12]. Fontaine and colleagues were among the first researchers to provide evidence that obese women were less frequently screened for cervical and breast cancer at the recommended intervals [13], [14]. In addition, the disabled population is well known as a particularly vulnerable population that is consistently underserved: they present substantial health disparities and are often under-screened [15]–[17]. Numerous studies have shown that screening rates are strongly influenced by the severity of the disability, with a gradient observed in the use of screening programs, including Pap tests and mammograms [18], [19].

Obesity and disability are often associated with each other [20]. There is a major disparity in weight between women with and without disabilities [15], [21]. Persons who have a preexisting physical impairment have been shown to be predisposed to being obese for a variety of reasons, including a low activity level [22], [23]. Increased weight may be more problematic among people living with disabilities than among the nondisabled population [24]. The combination of mobility disability and weight gain can result in a vicious cycle, posing additional health problems and disability-related limitations, thereby increasing the severity of the disability [24], [25]. Indeed, obesity can lead to mobility disability and personal care disability, most often from arthritic conditions [26], [27]. The relationship between obesity and disability is clearly complex; obesity is causal in some cases, whereas the disability may be the primary disorder in other cases.

Given the increasing prevalence of obesity and disabling conditions [28], [29] as well as their strong association, linking these two health priorities is essential. This study contributes to the literature by exploring the joint and separate effects of obesity and mobility limitation on participation in cervical and breast cancer screening programs among community-dwelling women in France.

## Materials and Methods

### Data Source

The source of the data used in this study was the Health and Disability Survey – Ordinary Household Section (Enquête Handicap Santé-Ménage, HSM, available at http://www.sante.gouv.fr/handicap-sante.html), which was performed from April to September of 2008 by the French National Institute of Statistics and Economic Studies (INSEE), and the French Head Office of Research, Studies, Evaluation and Statistics of the Social Affairs Ministry.

The HSM is a national cross-sectional survey that aims to measure the prevalence of various forms of disabling situations, applying concepts developed by the World Health Organization, to assess the need for aid and to measure the social disadvantages of disabled people. The new concepts of disability listed in the International Classification of Functioning, Disability and Health [30] (ICF) place the notions of ‘health’ and ‘disability’ in a new light. The ICF identifies three levels of human function: functioning at the levels of body parts (body level), the whole person (individual level) and the whole person in their complete environment (societal level). These levels, in turn, contain three domains of human function: body functions and structures, activities and participation. The term ‘disability’ is used to denote a decrement at each level, i.e., an impairment, functional limitation or restriction in participation. Thus, in the HSM survey, individuals were asked about their impairments (physical, sensory and/or cognitive), their functional limitations (mobility, sensory and/or cognitive) and social participation restrictions (access to the labor market, educational opportunities and leisure as well as aspects of their standards of living, familial or social network, general accessibility and/or experience with discrimination). They were also asked about their diseases, their use of healthcare and the different forms of aid they received or needed. Information about their socio-demographic characteristics was also collected.

The HSM sample was taken from the database of respondents to the filter survey *Everyday Life and Health Survey* (VQS), which was administered to approximately 140,000 households (260,000 individuals), across French territories (mainland France and overseas territories) in 2007 by mail, by telephone or face-to-face. The VQS is a filter survey intended to prepare the sample for an in-depth survey of people with physical deficiencies, functional limitations or, more generally, difficulties in accomplishing certain activities of daily life. Because people in situations of disability or dependence are relatively rare with respect to the general population, they must be over-represented in the sample of a survey that targets their characteristics and situation to provide sufficiently robust results. The VQS achieved this outcome by sending a short questionnaire to a large number of households that contains several questions requesting a description of each member of the household. These questions concern the existence or absence of a recognized disability, difficulties in accomplishing certain tasks and other related questions. According to their answers, people were classified into four levels of presumed disability severity, from 1 (no disability) to 4 (high level of disability). These groups are then used to build the selection strata for the main survey. For sample selection, randomisation involved a high sampling rate for the most severely disabled group and a low sampling rate for people without daily living restrictions (the largest group). Each of the resulting groups was allocated a specific sampling coefficient that increased with the probability or severity of the presumed handicap. Thus, of the 260,000 individuals from the VQS filter, 39,065 were selected for the HSM survey, and 29,954 answered the questionnaire, corresponding to a response rate of 77%. In total, the HSM database contains 29,931 questionnaires considered to be ‘complete.’ Each respondent was assigned a weight reflecting the probability of being investigated (depending on presumed disability severity and geographic area of residence) and answering the questionnaire. Final weights ensured that the data were representative of the French population living in households. The design of the survey is summarized in a flow chart published by Clémence Palazzo et al. (2012) [31].

All information was directly gathered by trained investigators using the computer-assisted interview (CAPI) format to collect data from people in their homes. When individuals were unable to respond to the questionnaire by themselves, a proxy was asked to provide help; thus, all responses were self-reported or, in some cases, proxy-reported.

### Ethics

This study was planned as a research project. All precautions were taken by the INSEE to ensure anonymity of the data. This study was declared of public interest by the CNIS (Conseil National d'Information Statistique) and was approved by the CNIL (Commission Nationale de l'Informatique et des Libertés, French law no. 78-17). According to the French law, written informed consent was not required for this type of study.

### Study Participants

**Figure 1:**
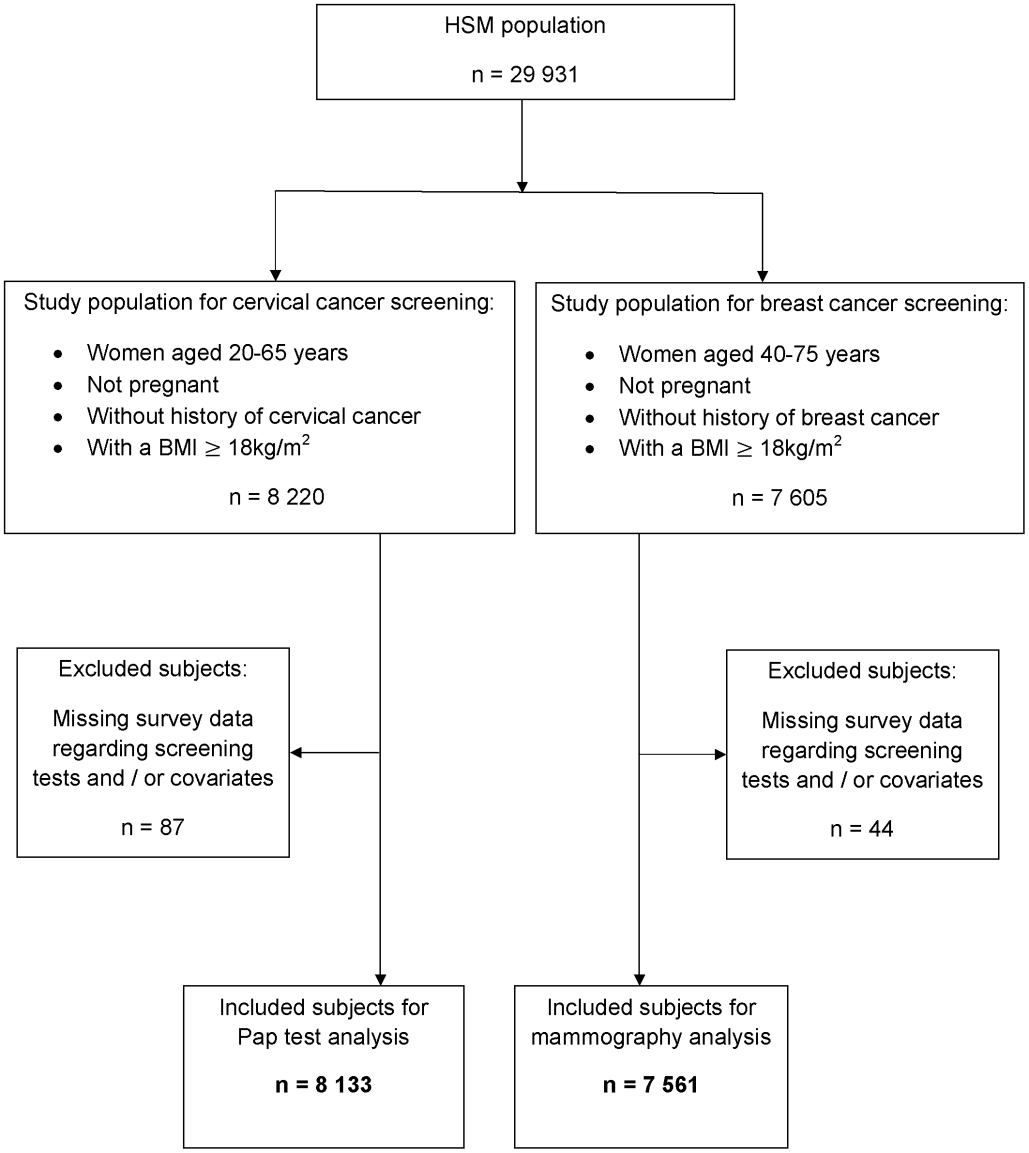
Flow chart illustrating the selection of study populations for cervical and breast cancer screening from the Health and Disability Survey - Household Section (HSM).

From the HSM population, we included all women aged 20–65 years for cervical cancer screening and those aged 40–75 years for breast cancer screening, as it is recommended by the European Union Council [32] and the U.S Preventive Services Task Force [33]. None of the participants were currently pregnant, and none had a history of cervical or breast cancer. Subjects with missing survey data regarding screening tests or covariates were excluded from the analysis. We also excluded underweight women (BMI<18 kg/m^2^). Ultimately, two groups were established: the group for cervical cancer screening analysis (n = 8,133) and the group for breast cancer screening analysis (n = 7,561) (Figure 1).

### Statistical Analysis

Descriptive analyses of the two groups were performed in terms of obesity, mobility limitation level, socio-demographic status, health and health care use variables. Chi-square tests were used to compare all characteristics between those who did and did not receive the screening test in each study group, with the exception of the mobility limitation level, for which t-tests were used. Finally, t-tests were performed to determine whether the mean of the mobility limitation level differed significantly by obesity status.

Odds ratios (ORs), adjusted odds ratios (AORs) and 95% confidence intervals (95% CIs) were estimated from weighted logistic regression models to analyze the association between body weight status, severity of disability and cancer screening use. First, univariate models were used to examine obesity and severity of disability separately in association with each outcome. Second, both obesity and severity of disability were included as the main effects in the models. Next, the models were adjusted for the socio-demographic, health and health care use variables. Lastly, interaction terms between obesity and the severity of disability were included in the models. Because obesity and disability are commonly linked, testing for the interaction enabled us to examine whether the effect of obesity differed based on disability level and vice versa. All analyses were conducted in 2013 using SAS 9.3 software.

### Outcome of interest

The outcomes of interest were the following: whether a Pap test was performed within the 3 years prior to the interview and whether a mammogram was performed within the 2 years prior to the interview.

### Explanatory variables

#### Obesity

Body mass index was calculated in kg/m^2^ from the self-reported body weight and height of each individual and dichotomized as obese (BMI≥30 kg/m^2^) or not obese (BMI<30 kg/m^2^).

#### Mobility limitation level

We constructed a continuous severity score, ranging from 0 to 27, by summing each respondent's self-reported level of difficulty (no difficulty = 0; some difficulty = 1; much difficulty = 2; unable to perform = 3) in performing each of the following nine tasks without any aid: (1) controlling stool and urine; (2) biting and chewing hard foods, such as an apple; (3) stooping, crouching and/or kneeling; (4) raising arms (for example, moving an object from an elevated place); (5) walking 500 meters on flat ground; (6) walking up and down a flight of stairs; (7) grasping or holding an object in their hands; (8) using hands and fingers with a normal level of dexterity (for example, turning on a faucet or using a pencil); and (9) lifting or carrying a bag as heavy as 5 kilograms for a distance of 10 meters. The choice of the items assessing the mobility limitations was based on the Nagi Disability Scale [34], and also inspired by previous studies [23], [25], [35], [36]. We verified the homogeneity of the items using Cronbach's alpha, and we obtained a value of 0.84, which corresponds to excellent internal consistency. The continuous severity score is hereafter termed the *disability score*. The Pearson's r correlation between this disability score and the screening use percentages were -0.76 for Pap test use and −0.87 for mammogram use (p<0.0001). This result indicates strong negative linear relationships between the disability score and the two cancer screening rates. We therefore chose to retain the score as a continuous variable in our models.

#### Socio-demographic, health and health care use variables

The socio-demographic variables included were age (categorized in 10-year age groups); marital status (single/married/divorced/widowed); level of education (non-high school graduate/high school graduate); and employment status (employed/retired/unemployed). The latter two variables were used as proxies for socio-economic status. Health and health care use variables included having at least one chronic disease (i.e., when the course of the disease lasted for more than 6 months) (yes/no) and visit(s) to a general practitioner in the last year (yes/no). These variables were primarily selected on the basis of earlier studies [9], [37].

## Results

### Characteristics of the study participants

Table 1 summarizes the characteristics of the two groups. The chi-square analysis showed that all variables were significantly associated with screening use (Table 2). Women with a lower level of education and those who were unmarried or unemployed were less likely to receive a Pap test in the past 3 years or a mammogram in the past 2 years. Women who have visited their general practitioner were more likely to receive these screening tests. The presence of a chronic disease was associated with less Pap tests but more mammograms. We also observed a significant peak in Pap test use in women between 30 and 40 years of age and a peak in mammogram use in women between 50 and 65 years of age. BMI was significantly associated with screening use: obese women reported lower rates of screening use compared with women with a BMI under 30 kg/m^2^. Concerning the disability score, the mean score for those who received screening tests was significantly lower than the mean score for those who did not receive screening for both cervical and breast cancer. Comparisons of disability score means by obesity status are given in table 3; these results show that the mean disability score was significantly higher in obese women than in non-obese women in the two groups studied.

**Table 1:** Characteristics of study participants.

		Group for cervical cancer screening analysis^a^		Group for breast cancer screening analysis^b^	
Characteristics		*n*	*%*	*n*	*%*
		8,133	100.0	7,561	100.0
**Age**					
	20–30	890	10.9	_	_
	30–40	1,341	16.5	_	_
	40–50	2,048	25.2	2,029	26.8
	50–65	3,854	47.4	3,732	49.4
	65–75	_	_	1,800	23.8
**Educational level**					
	Non-high school graduate	5,679	69.8	6,024	79.7
	High school graduate	2,454	30.2	1,537	20.3
**Marital status**					
	Married	4,390	54.0	4,450	58.9
	Single	2,354	28.9	1,171	15.5
	Divorced	972	12.0	1,005	13.3
	Widowed	417	5.1	935	12.4
**Employment status**					
	Employed	3,910	48.1	2,588	34.2
	Retired	984	12.1	2,530	33.5
	Unemployed	3,239	39.8	2,443	32.3
**Chronic disease(s)**					
	Yes	5,034	61.9	5,446	72.0
	No	3,099	38.1	2,115	28.0
**Visit(s) to the GP in the last year**					
	Yes	7,486	92.0	7,063	93.4
	No	647	8.0	498	6.6
**Obesity**					
	Yes (BMI≥30)	1,707	21.0	1,854	24.5
	No (BMI<30)	6,426	79.0	5,707	75.5
**Disability score**					
	Mean ± S.D	2,76±4,43		4,14±5,28	
	= 0	4,137	50.9	2,727	36.1
	≥1	3,996	49.1	4,834	63.9

Notes. GP =  General Practitioner; BMI =  Body Mass Index.

a Women aged 20–65 years, not pregnant and without history of cervical cancer.

b Women aged 40–75 years, not pregnant and without history of breast cancer.

**Table 2:** Comparison of characteristics of study participants between those who did and did not receive the screening test^a^.

		Individuals who received a Pap test within 3-years^b^		Individuals who received a mammogram within 2-years^c^	
Characteristics		*n*	*(row) %*	*n*	*(row) %*
		5,795	71.3	5,140	68.0
**Age**					
	20–30	600	67.4	_	_
	30–40	1,075	80.2	_	_
	40–50	1,582	77.3	1,073	52.9
	50–65	2,538	65.9	2,866	76.8
	65–75	_	_	1,201	66.7
			*p<0.0001*		*p<0.0001*
**Educational level**					
	Non-high school graduate	3,791	66.8	3,997	66.4
	High school graduate	2,004	81.7	1,143	74.4
			*p<0.0001*		*p<0.0001*
**Marital status**					
	Married	3,299	75.2	3,216	72.3
	Single	1,560	66.3	661	56.5
	Divorced	693	71.3	694	69.1
	Widowed	243	58.3	569	60.9
			*p<0.0001*		*p<0.0001*
**Employment status**					
	Employed	3,168	81.0	1,809	69.9
	Retired	607	61.7	1,810	71.5
	Unemployed	2,020	62.4	1,521	62.3
			*p<0.0001*		*p<0.0001*
**Chronic disease(s)**					
	Yes	3,436	68.3	3,737	68.6
	No	2,359	76.1	1,403	66.3
			*p<0.0001*		*p = 0.0461*
**Visit(s) to the GP in the last year**					
	Yes	5,389	72.0	4,880	69.1
	No	406	62.8	260	52.2
			*p<0.0001*		*p<0.0001*
**Obesity**					
	Yes (BMI≥30)	1,041	61.0	1,183	63.3
	No (BMI<30)	4,754	74.0	3,957	69.8
			*p<0.0001*		*p<0.0001*
**Disability score**					
	Mean ± S.D	2,29±3,98		3,81±4,94	
			*p<0.0001* d		*p<0.0001* d
	= 0	3,211	77.6	1,908	70.0
	≥1	2,584	64.7	3,232	66.3
			*p<0.0001*		*p = 0.0054*

Notes. GP =  General Practitioner; BMI =  Body Mass Index.

a Bivariate analyses were performed by chi-square testing comparing characteristics between those who did and did not receive the screening test.

b Group for cervical cancer screening analysis: women aged 20–65 years, not pregnant and without history of cervical cancer.

c Group for breast cancer screening analysis: women aged 40–75 years, not pregnant and without history of breast cancer.

d A t-test was performed to determine whether the mean score for those who received the screening test differed significantly from the mean score for those who didn't receive it, in each study group.

**Table 3:** Comparison of disability score means by obesity status^a^.

	Not obese	Obese
	(BMI<30)	(BMI≥30)
Group for cervical cancer screening analysis	2,24±4,01	4,82±5,32
**Disability score**		
Mean ± S.D		
Group for breast cancer screening analysis	3,47±4,91	6,19±5,75
**Disability score**		
Mean ± S.D		

Notes. BMI =  Body Mass Index.

a T-tests were performed to determine whether or the mean score differed significantly by BMI categories, in each study group. All tests were significant - *P<.0001*.

### Logistic Regression Models

#### Univariate and bivariate models

Table 4 summarizes the results of the logistic regressions for the univariate and bivariate models. The results were very similar for the two models. A significant gradient in screening use with respect to obesity and disability score was observed. Relative to the non-obese women, obese women were less likely to report a Pap test in the past 3 years or a mammogram in the past 2 years. Additionally, as the disability score increased, the likelihood of reporting a Pap test or a mammogram decreased.

**Table 4:** Weighted logistic regressions^a^ on the use of Pap tests and mammograms: Univariate and Bivariate models.

	Pap test within 3 years^b^		Mammogram within 2 years^c^	
	n = 8 133		n = 7 561	
Variables	OR	95% CI	OR	95% CI
		**Univariate Model**		
**Obesity**				
Yes (BMI≥30)	0,59***	0,51–0,59	0,75***	0,65–0,87
No (BMI<30)	Referent		Referent	
**Disability score**	0,91***	0,89–0,93	0,97**	0,95–0,98
		**Bivariate Model**		
**Obesity**				
Yes (BMI≥30)	Referent		Referent	
No (BMI<30)	0,68***	0,58–0,78	0,79**	0,68–0,91
**Disability score**	0,92***	0,90–0,94	0,97**	0,96–0,99

*Notes.* OR =  Odds Ratios; CI =  Confidence Intervals; BMI =  Body Mass Index.

a Analysis were performed on probability-weighted sample data with SAS 9.3 software.

b Women aged 20–65 years, not pregnant and without history of cervical cancer.

c Women aged 40–75 years, not pregnant and without history of breast cancer.

*P<.1;

**P<.05;

***P<.0001.

#### Multivariate model

In the model that was adjusted for covariates, we found significant negative associations between obesity and Pap testing (AOR = 0.76; 95% CI: 0.65 to 0.89), between obesity and mammograms (AOR = 0.77; 95% CI: 0.66 to 0.91), between disability score and Pap testing (AOR = 0.96; 95% CI: 0.94 to 0.98) and between disability score and mammograms (AOR = 0.95; 95% CI: 0.94 to 0.97). To more meaningfully interpret the latter two AORs, each time the disability score increased by 5 points, the odds of having a Pap test in the past 3 years decreased by 20% ((1–0.96)×100×5) and the odds of having a mammogram in the past 2 years decreased by 25% ((1–0.95)×100×5) (Table 5).

**Table 5:** Weighted logistic regressions^a^ on the use of Pap tests and mammograms: Multivariable model.

	Pap test within 3 years^b^		Mammogram within 2 years^c^	
	n = 8 133		n = 7 561	
Variables	AOR	95% CI	AOR	95% CI
**Age**				
20–30	0.77**	0.64–0.94	_	_
30–40	1.47***	1.22–1.78	_	_
40–50	Referent		Referent	
50–65	0.70***	0.60–0.82	3.78***	3.25–4.40
65–75	_	_	2.78***	2.20–3.51
**Educational level**				
Non-high school graduate	0.62***	0.55–0.70	0.66***	0.57–0.75
High school graduate	Referent		Referent	
**Marital status**				
Married	Referent		Referent	
Single	0.67***	0.58–0.78	0.51***	0.43–0.60
Divorced	0.78**	0.65–0.95	0.84*	0.70–1.01
Widowed	0.79*	0.60–1.02	0.55***	0.46–0.67
**Employment status**				
Employed	Referent		Referent	
Retired	0.52***	0.43–0.64	0.99	0.78–1.26
Unemployed	0.46***	0.41–0.52	0.70***	0.59–0.82
**Chronic disease(s)**				
Yes	1.07	0.94–1.21	1.24**	1.09–1.41
No	Referent		Referent	
**Visit(s) to the GP in the last year**				
Yes	Referent		Referent	
No	0.50***	0.43–0.59	0.44***	0.36–0.53
**Obesity**				
Yes (BMI≥30)	0.76**	0.65–0.89	0.77**	0.66–0.91
No (BMI<30)	Referent		Referent	
**Disability score**	0.96**	0.94–0.98	0.95***	0.94–0.97

*Notes.* AOR =  Adjusted Odds Ratios; CI =  Confidence Intervals; GP =  General Practitioner; BMI =  Body Mass Index.

a Analysis were performed on probability-weighted sample data adjusted for potential confounders with SAS 9.3 software.

b Women aged 20–65 years, not pregnant and without history of cervical cancer.

c Women aged 40–75 years, not pregnant and without history of breast cancer.

*P<.1;

**P<.05;

***P<.0001.

#### Models with interactions

**Figure 2:**
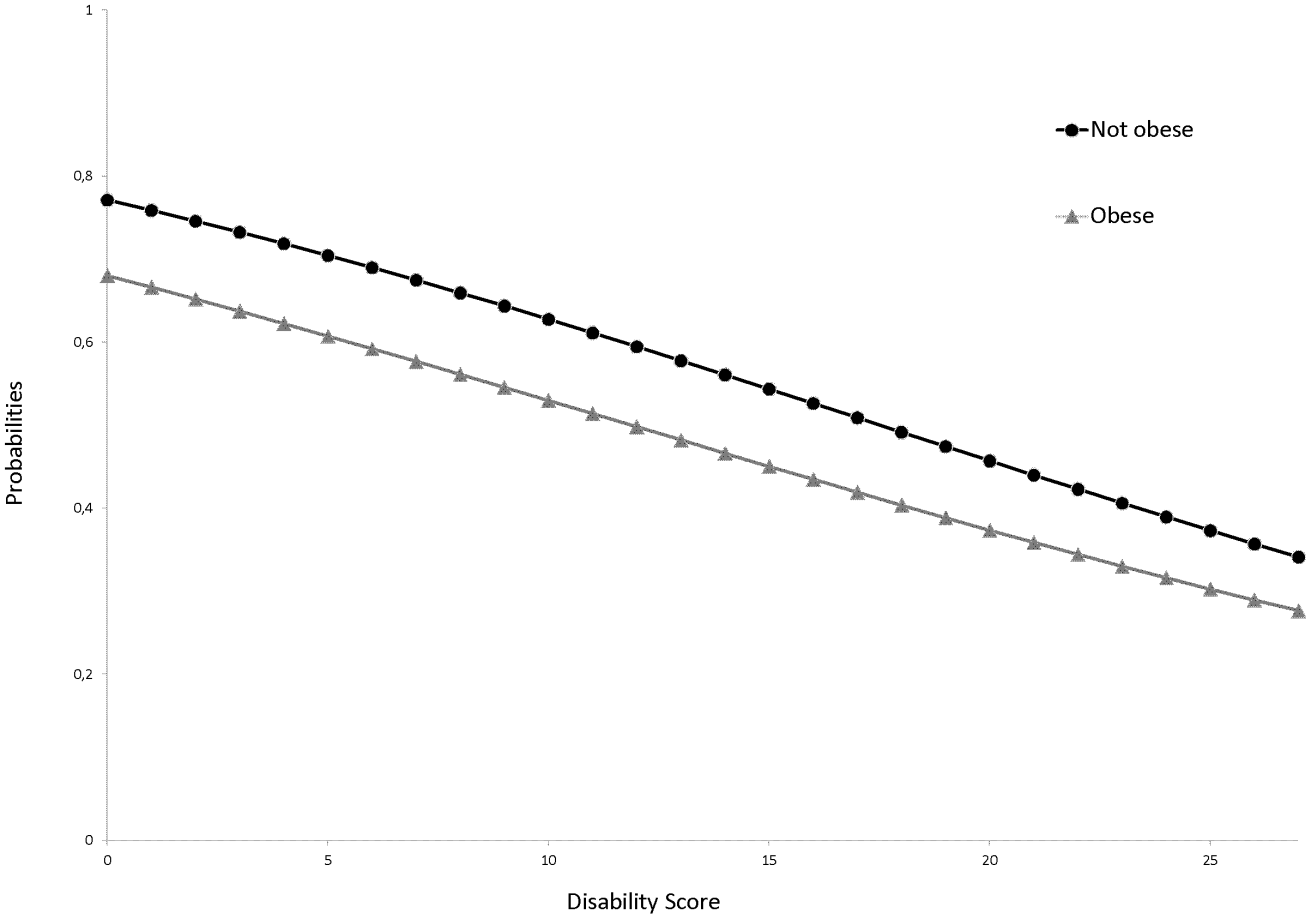
Predicted probabilities of having received a Pap test within the past 3 years: model with an interaction term between obesity and disability score.

**Figure 3:**
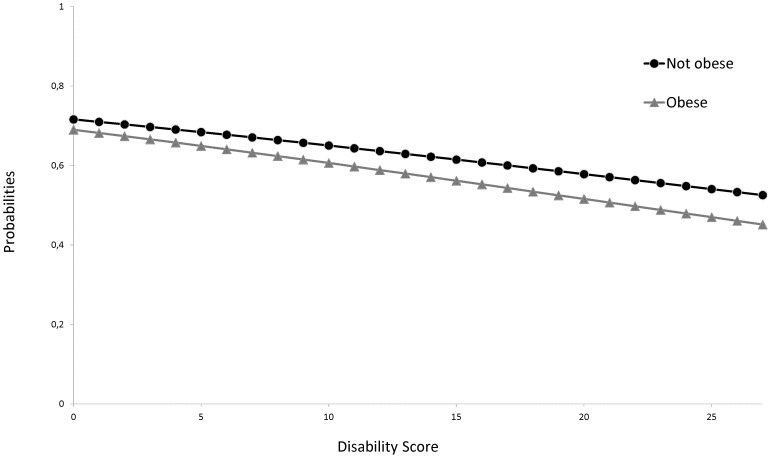
Predicted probabilities of having received a mammogram within the past 2 years: model with an interaction term between obesity and disability score.

We did not find any significant interaction between obesity and disability score. The slopes of the logistic curves did not differ based on obesity status. The probability of having received a Pap test or a mammogram decreased similarly with the disability score in obese and non-obese women (Figures 2 and 3).

## Discussion

To the best of our knowledge, this study is the first to investigate the relative contributions of obesity and mobility disability to both cervical and breast cancer screening. Using a large population-based sample from the French national *Health and Disability Survey*, our analysis showed that obesity and mobility limitation are independently associated with a lower likelihood of cervical and breast cancer screening. The higher the BMI or disability score is, the lower the likelihood is of Pap test and mammogram use. There were no significant interaction effects.

Our results also confirm several significant associations usually found in the literature [6], [8], [9] between socio-demographic, health and health care use-related characteristics and cancer screening use. Indeed, according to regression models, having a higher educational level, being married, being employed, having at least one chronic disease and having visited a GP in the past year were associated with a higher likelihood of screening.

To reduce cancer-related mortality, the political focal point of recent decades has been to improve access to routine screening. To shift the focus in this manner and to plan appropriate public health programs, it is important to clearly identify existing inequalities and the reasons for these inequalities.

Our results first highlight the differences in access to routine screening. Compared with 74% of non-obese women, only 61% of obese women reported having a Pap test within the past 3 years. Among women with a disability score of 0, 78% received a Pap test within the past 3 years, whereas only 65% of those with a disability score greater than or equal to 1 received a Pap test. The differences were not as pronounced for breast cancer screening but remained significant. These results are highly important from a public health perspective, mostly as we know now that cancer incidence and mortality can be significantly reduced if cases are detected and treated early [2]. For example, it has been shown that mammography can reduce breast cancer mortality by 20 to 30% when the screening coverage is over 70% [38]. A Pap testing coverage of 80% would decrease the cervical cancer incidence by 2.5% per year [39].

Second, in addition to strengthening the already well-known links between the level of limitation, obesity and the lower rate of screening, these findings also help us to better understand the complex relationships among these factors. According to our results, obesity and disability appear to have similar but independent effects on the rate of screening test participation. It is already well known that functional limitations as such constitute a barrier to cancer screening use [11], [16]. People with mobility limitations encounter logistic and/or architectural obstacles such as access to buildings, machines and examination tables [11], [40]. A telephone survey [41] recently conducted among 256 physicians in the US confirmed a lack of accessibility to care—especially gynecological care—among people with a mobility impairment. In the current study, we also showed that obesity as such constitutes a barrier to cancer screening use that is independent of the functional limitations commonly found in obese people, which are usually mentioned in the literature to explain the well-known association between obesity and decreased rate of cancer screening [42], [43]. The literature provides some interpretations of these results. According to Fontaine and colleagues [13], [14], apart from the equipment- or facility-related barriers due to mobility limitations, attitudinal obstacles are also given as reasons for the lower frequency of preventive care among people with obesity. These obstacles include competing demands for the management of other obesity-related chronic conditions; the fear of discomfort and pain from procedures; anxiety regarding physical privacy, ridicule or reproach due to excess weight; and a low perceived risk. Findings from these early studies have been confirmed by a number of subsequent studies [44], [45]. For example, vaginal speculum examinations and mammograms have been reported to potentially be more difficult, painful or time-consuming for obese women, which may lead to deferral of the exam. Another recent study [46] based on qualitative data reported that disrespectful treatment, embarrassment and negative attitudes of providers as well as insensitive comments about weight are all barriers to routine gynecologic cancer screening.

Finally, we showed that there was no interaction effect between obesity and disability score. These findings suggest that, although essential, addressing limitation-related barriers by modifying environmental factors is not sufficient to achieve a high level of participation in screening programs among individuals in these vulnerable populations. Governments from developed countries have enacted laws and established norms that require adaptation of infrastructure and medical equipment to allow access for people with functional limitations [47]. There are also international standards that address the accessibility of buildings, such as *ISO/TR 9527:1994, Building construction — Needs of disabled people in buildings — Design guidelines*. At a national level, many European Union member states have regulations and standards concerning buildings and transport. They have accessibility provisions in their legislation (generally anti-discrimination legislation), such as the Disability Discrimination Act (DDA) in the UK, the Liondau law in Spain (law 51/2003 on equal opportunities, non-discrimination and universal accessibility for people with disabilities) and the law of 11 February 2005 in France (law on equal rights and opportunities, participation and citizenship of people with disabilities).

However, current health policies do not view obesity alone as a barrier to screening participation. Our finding reveals the importance of raising awareness among medical personnel who provide screening tests and the importance of fostering self-confidence among obese individuals. Doctors or other health care professionals may need to explicitly talk to their obese patients about the importance of following screening recommendations, just as they do with their non-obese patients, if not to a greater extent. This is especially important because obese women are at increased risk of developing these cancers [48].

This study has limitations that are common to this type of survey. The main limitation is that data were self-reported and not physician-confirmed, which is likely to be accurate for disability assessment but may lack accuracy for screening. Self-reported screening behaviors from national surveys often overestimate screening use [49]. If this was the case, it would strengthen our results. Second, height and weight, which are the metrics used to calculate BMI, were not clinically evaluated. Previous studies have shown that weight is often under-reported, especially in overweight female populations, whereas height is generally over-reported, leading to an underestimation of BMI and a misclassification in BMI categories [50]. This phenomenon is partially explained by social desirability, which can be further influenced by the method of data collection. Indeed, a Canadian study [51] showed that obesity prevalence in a group that was surveyed face-to-face was significantly higher than that in a group that was surveyed by phone. We can assume that this weight and height reporting bias was attenuated in our present study. Furthermore, an underestimation of BMI could also work in favor of the results of the current study in the sense that it most likely led to an underestimation of the highlighted association between obesity and cancer screening. Another limitation of the present study concerns individuals who were excluded because they did not answer the screening questions or other questions related to the independent variables. This exclusion could constitute a selection bias; however, it affected a very small number of individuals compared with the overall sample.

## Conclusions

Our findings are especially intriguing because they highlight a lack of preventive care among women included in a healthcare system in which women are widely encouraged to be screened through national screening programs, recommendations, reminders, ease of payment and reimbursements. Policies should provide comprehensive strategies with a focus on improving the attitudes of providers, support for women and encouragement to follow cancer screening interval recommendations, particularly in these vulnerable and high-risk populations of obese women with disabilities. Protective outreach and follow-up are necessary to reduce inequalities and thus to reduce health disparities.
